# Association of Maternal Neurodevelopmental Risk Alleles With Early-Life Exposures

**DOI:** 10.1001/jamapsychiatry.2019.0774

**Published:** 2019-05-01

**Authors:** Beate Leppert, Alexandra Havdahl, Lucy Riglin, Hannah J. Jones, Jie Zheng, George Davey Smith, Kate Tilling, Anita Thapar, Ted Reichborn-Kjennerud, Evie Stergiakouli

**Affiliations:** 1 MRC (Medical Research Council) Integrative Epidemiology Unit, University of Bristol, Bristol, United Kingdom; 2 Population Health Sciences, Bristol Medical School, University of Bristol, Bristol, United Kingdom; 3 Nic Waals Institute, Lovisenberg Diaconal Hospital, Oslo, Norway; 4 Department of Mental Disorders, Norwegian Institute of Public Health, Oslo, Norway; 5 MRC Centre for Neuropsychiatric Genetics and Genomics, Cardiff University, Cardiff, United Kingdom; 6 National Institute for Health Research Biomedical Research Centre, University Hospitals Bristol NHS (National Health Service) Foundation Trust and the University of Bristol, Bristol, United Kingdom; 7 Institute of Clinical Medicine, University of Oslo, Oslo, Norway; 8 School of Oral and Dental Sciences, University of Bristol, Bristol, United Kingdom

## Abstract

**Importance:**

Early-life exposures, such as prenatal maternal lifestyle, illnesses, nutritional deficiencies, toxin levels, and adverse birth events, have long been considered potential risk factors for neurodevelopmental disorders in offspring. However, maternal genetic factors could be confounding the association between early-life exposures and neurodevelopmental outcomes in offspring, which makes inferring a causal relationship problematic.

**Objective:**

To test whether maternal polygenic risk scores (PRSs) for neurodevelopmental disorders were associated with early-life exposures previously linked to the disorders.

**Design, Setting, and Participants:**

In this UK population-based cohort study, 7921 mothers with genotype data from the Avon Longitudinal Study of Parents and Children (ALSPAC) underwent testing for association of maternal PRS for attention-deficit/hyperactivity disorder (ADHD PRS), autism spectrum disorder (ASD PRS), and schizophrenia (SCZ PRS) with 32 early-life exposures. ALSPAC data collection began September 6, 1990, and is ongoing. Data were analyzed for the current study from April 1 to September 1, 2018.

**Exposures:**

Maternal ADHD PRS, ASD PRS, and SCZ PRS were calculated using discovery effect size estimates from the largest available genome-wide association study and a significance threshold of *P* < .05.

**Main Outcomes and Measures:**

Outcomes measured included questionnaire data on maternal lifestyle and behavior (eg, smoking, alcohol consumption, body mass index, and maternal age), maternal use of nutritional supplements and medications in pregnancy (eg, acetaminophen, iron, zinc, folic acid, and vitamins), maternal illnesses (eg, diabetes, hypertension, rheumatism, psoriasis, and depression), and perinatal factors (eg, birth weight, preterm birth, and cesarean delivery).

**Results:**

Maternal PRSs were available from 7921 mothers (mean [SD] age, 28.5 [4.8] years). The ADHD PRS was associated with multiple prenatal factors, including infections (odds ratio [OR], 1.11; 95% CI, 1.04-1.18), use of acetaminophen during late pregnancy (OR, 1.11; 95% CI, 1.04-1.18), lower blood levels of mercury (β coefficient, −0.06; 95% CI, −0.11 to −0.02), and higher blood levels of cadmium (β coefficient, 0.07; 95% CI, 0.05-0.09). Little evidence of associations between ASD PRS or SCZ PRS and prenatal factors or of association between any of the PRSs and adverse birth events was found. Sensitivity analyses revealed consistent results.

**Conclusions and Relevance:**

These findings suggest that maternal risk alleles for neurodevelopmental disorders, primarily ADHD, are associated with some pregnancy-related exposures. These findings highlight the need to carefully account for potential genetic confounding and triangulate evidence from different approaches when assessing the effects of prenatal exposures on neurodevelopmental disorders in offspring.

## Key Points

**Question:**

Are maternal polygenic risk scores for neurodevelopmental disorders associated with early-life exposures?

**Findings:**

In this population-based cohort study of 7921 mothers, polygenic risk score for attention-deficit/hyperactivity disorder was associated with a range of early-life exposures linked to neurodevelopmental disorders in offspring. Polygenic risk scores for autism spectrum disorder and schizophrenia showed little evidence association with of early-life exposures.

**Meaning:**

The findings suggest that mothers at high genetic risk for attention-deficit/hyperactivity disorder may also be at increased risk for some adverse pregnancy exposures, and future studies should triangulate evidence from different causally informative approaches.

## Teaser

This population-based cohort study assesses whether maternal polygenic risk scores for neurodevelopmental disorders are associated with early-life exposures previously linked to the disorders in women with available genotype data.

## Introduction

Neurodevelopmental disorders involve atypical brain development affecting domains such as language, motor skills, social communication, attention, activity regulation, and learning.^1^ This group of disorders includes attention-deficit/hyperactivity disorder (ADHD) and autism spectrum disorder (ASD) and is sometimes broadened to encompass schizophrenia (SCZ) because its onset is often preceded by neurodevelopmental impairments. Neurodevelopmental disorders are highly heritable (60%-90%),^2,3^ although they are associated with a combination of multiple genetic, environmental, and stochastic factors.^4^

Prenatal and perinatal exposures to adversities have long been considered to be possible risk factors for neurodevelopmental disorders and are biologically plausible as causal risks because they occur during a sensitive time in neurodevelopment.^5^ Prenatal factors linked with neurodevelopmental disorders include smoking,^6,7,8^ alcohol intake,^9^ nutritional deficiencies (eg, vitamin D),^7^ poor nutrition,^10,11^ nonuse of nutritional supplements (eg, folic acid), medication use (eg, acetaminophen [paracetamol], antidepressants),^12,13,14^ prepregnancy body mass index (BMI) (calculated as weight in kilograms divided by height in meters squared),^15^ age at delivery,^16^ metabolic disorders (eg, diabetes, preeclampsia),^17^ immune-related conditions (eg, infection, autoimmune diseases),^18,19^ depression,^10,11^ stressful life events,^9,20^ and toxin levels (eg, mercury, lead).^21,22^ Adverse perinatal factors (eg, low birth weight, prematurity, cesarean delivery) have been shown to be associated with all 3 neurodevelopmental disorders.^23,24,25^ However, with some exceptions,^10,11^ most of these studies were observational and are therefore not appropriate for assessing causality.^26^

Confounding by sometimes unknown lifestyle, socioeconomic, or genetic factors can lead to apparent associations between early-life factors and neurodevelopmental outcomes that are not causal. Potential genetic confounding is particularly important for factors such as maternal smoking, which is known to be associated with maternal genotype.^27,28^ Other factors such as birth weight are associated with maternal and fetal genotypes.^29^ If genetic factors are independently associated with neurodevelopmental disorders and early-life exposures (horizontal pleiotropy), then associations could arise for noncausal reasons. For example, of 12 studies using different causally informative designs, such as discordant sibling pairs,^30^ maternal vs paternal exposures,^31^ an assisted conception cohort,^32^ and children of twins,^33^ 11 showed that maternal smoking in pregnancy does not appear to have a causal effect on ADHD, although it has an effect on lowering birth weight.^34^ However, such designs have not been able to address the full range of prenatal and perinatal risks.

Genome-wide association studies (GWAS) suggest that genetic liability to neurodevelopmental disorders is in part conferred by a large number of common (present in >1% of the population) single-nucleotide polymorphisms (SNPs).^35,36,37^ These SNPs can be used to generate composite measures called polygenic risk scores (PRSs),^38^ which have been shown to be associated with a range of phenotypes in the general population for neurodevelopmental disorders.^39,40^ In this study, we used a large population-based pregnancy cohort from the United Kingdom to investigate whether PRSs for ADHD (ADHD PRS), ASD (ASD PRS), and schizophrenia (SCZ PRS) are associated with early-life exposures linked to these disorders.^9,20,41^

## Methods

### Avon Longitudinal Study of Parents and Children Study Data

The ongoing prospective Avon Longitudinal Study of Parents and Children (ALSPAC) initially recruited 14 541 pregnant women living in Avon, United Kingdom, with expected delivery dates from April 1991 to December 1992 and with 13 988 children alive at year 1. Of those, 10 015 mothers and 9912 children underwent genotyping using 2 platforms (Human660W-quad and Human Hap550-quad [Illumina], respectively). After standard quality control, the resulting data set included 7921 unrelated mothers and 7975 unrelated children of European ancestry. Detailed procedures for quality control have been published previously.^42^ Detailed information on the health and lifestyles of mothers and children was collected from regular clinical visits and self-administrated questionnaires. A detailed description of the cohort has been published previously.^43,44^ The study website contains a fully searchable data dictionary and variable search tool.^45^ Ethical approval for the study was obtained from the ALSPAC Ethics and Law Committee and the local research ethics committees.^46^ All participants provided written informed consent. ALSPAC data collection began September 6, 1990, and is ongoing. Data were analyzed for the current study from April 1 to September 1, 2018.

### Polygenic Risk Score

Polygenic risk scores were calculated using PRSice, version 1.25 (Statistical Genetics Unit, King’s College London) and PLINK, version 1.9,^47^ as the weighted mean number of disorder risk alleles in approximate linkage equilibrium (*R*^2^ < 0.1 within 1000-kilobase distance), as described previously.^48^ Risk alleles for ADHD and ASD were defined as those identified in the most recent combined Psychiatric Genomics Consortium and Lundbeck Foundation Initiative for Integrative Psychiatric Research (iPSYCH) analysis of case-control GWAS^35,36^ at a significance threshold of *P* < .05 to maximize phenotypic variance. Risk alleles for schizophrenia were identified by the Psychiatric Genomics Consortium meta-analysis.^37^ For sensitivity analysis, PRSs were derived using various *P* values. The number of SNPs included for each threshold and correlations among PRSs are provided in eTables 1 and 2 in the Supplement.

### Outcome Measures

#### Prenatal Factors Related to Maternal Lifestyle

Maternal smoking and alcohol consumption were assessed by self-report during the first and third trimesters; the participants were asked whether they had smoked or consumed alcohol in the past 3 months. The 2 measurements were used to classify smoking and alcohol consumption during pregnancy as any time vs never. Binge drinking was defined as having consumed the equivalent of more than 4 drinks per day at least once during the past 4 weeks.^49^ Maternal BMI was derived from self-reported prepregnancy height and weight. Maternal age was obtained at recruitment.

#### Maternal Use of Nutritional Supplements and Medications in Pregnancy

The use of antidepressants and the intake of iron, zinc, folic acid, or vitamin supplements were categorized as never vs ever based on maternal report in the first and third trimesters. Use of acetaminophen was maternally reported for the first (week 18) and the second (week 32) halves of pregnancy.

#### Maternal Illnesses and Conditions

Stressful life events scores were calculated based on maternal report of whether participants had experienced 18 different stressful life events during the first and third trimesters of their pregnancy.^50^ Severe depression, diabetes, hypertension, rheumatism, and psoriasis were categorized as ever vs never experienced. In addition, mothers were asked if they experienced preeclampsia, infections, or bleeding during pregnancy.

#### Biomarkers of Nutritional Status and Toxin Exposure in Pregnancy

Blood levels of vitamin D (25-hydroxyvitamin D), selenium, mercury, cadmium, and lead were measured in maternal blood samples during pregnancy, as described elsewhere.^51,52^ All of these measures were log-transformed.

#### Perinatal Factors and Conditions

Adverse birth events included low birth weight, preterm delivery, cesarean delivery, hypoxia, and Apgar scores at 1 and 5 minutes after birth, and were obtained from obstetric records. Low birth weight was defined as less than 2500 g; preterm delivery, born before 37 weeks’ gestation. The Apgar score was dichotomized as healthy when 7 or greater and adverse when less than 7.^53^ Mothers were asked if they initiated breastfeeding their child in the first month after birth.

### Statistical Analysis

Data were analyzed from April 1 to September 1, 2018. Associations of early-life exposures with maternal ADHD PRS, ASD PRS, or SCZ PRS were assessed using general linear models for continuous outcomes and general linear models with logit-link function and Poisson distribution for binary outcomes in Stata software, version 15.1 (StataCorp). Ten population stratification principal components derived from unrelated individuals using the Eigenstrat method^54^ were included as covariates in all analyses. In addition, the perinatal factors were tested for association with child ADHD PRS, ASD PRS, and SCZ PRS after adjusting for sex and 10 principal components because perinatal factors can be associated with maternal or child genetic risk. Effect estimates are presented per 1-SD increase in PRS.

To correct for multiple testing of 32 early-life exposures, the number of independent tests was determined based on the number of principal components that explained 80% of covariance between the early-life exposures in a principal component analysis (eMethods and eTable 3 in the Supplement). We concluded that 21 independent tests were performed, leading to a multiple testing–corrected *P* < .0024 (.05/21), which is slightly less conservative than the Bonferroni-corrected *P* < .0016 (.05/32) (assuming that all tests are independent).

As a sensitivity analysis, we performed an inverse probability weighting on missing maternal genetic data (eMethods in the Supplement) because ADHD PRS and SCZ PRS were associated with attrition in the ALSPAC core study.^55^ A comparison between the study samples with (n = 7486) and without (n = 6307) genetic data suggests that the groups differ in some of the analyzed factors (eMethods and eTable 4 in the Supplement).

## Results

### Associations Between PRSs and Early-Life Exposures

#### Factors Associated With Maternal Lifestyle

Descriptive characteristics of the sample are presented in Table 1. Mean (SD) age of mothers of the ALSPAC study sample was 28.5 (4.8) years; mean (SD) BMI, 22.9 (3.8). Maternal PRSs for neurodevelopmental disorders showed evidence of associations with smoking (ADHD PRS and SCZ PRS), use of acetaminophen (ADHD PRS), prepregnancy BMI (ADHD PRS and SCZ PRS), and age at delivery (ADHD PRS) (Table 2). A 1-SD increase in maternal ADHD PRS was associated with a 27% increase of smoking during pregnancy (odds ratio [OR], 1.27; 95% CI, 1.19-1.35); a 1-SD increase in maternal SCZ PRS, with an 11% increase of smoking during pregnancy (OR, 1.11; 95% CI, 1.04-1.18). Maternal ADHD PRS was associated with younger maternal age at delivery (β coefficient, −0.42; 95% CI, 0.53-0.31) (similar results were found for associations between PRSs and categorically defined age groups in eTable 5 in the Supplement). Association with maternal prepregnancy BMI occurred in opposite directions for ADHD PRS (β coefficient, 0.27; 95% CI, 0.18-0.36) and SCZ PRS (β coefficient, −0.16; 95% CI, −0.26 to −0.07). Examining associations at different times, we found evidence of an association between ADHD PRS and use of acetaminophen during the second half of pregnancy, such that a 1-SD increase in ADHD PRS was associated with an 11% increase in the odds of taking acetaminophen (OR, 1.11; 95% CI, 1.04-1.18).

**Table 1.** Sample Description

Exposure	Total No. of Participants	Participants With Exposure
Smoking, No. (%)		
During first trimester	7330	1620 (22.1)
During third trimester	6787	1157 (17.0)
Alcohol consumption, No. (%)		
During first trimester	7276	4051 (55.7)
During third trimester	4355	1526 (35.0)
Binge drinking during first trimester, No. (%)	7263	1181 (16.3)
Any nutritional supplements in pregnancy, No. (%)		
Iron	7270	1541 (21.2)
Zinc	7270	92 (1.3)
Folic acid	7263	688 (9.5)
Vitamins	7253	1190 (16.4)
Acetaminophen, No. (%)		
Early pregnancy	7142	3838 (53.7)
Late pregnancy	6748	2888 (42.8)
Any antidepressants in pregnancy, No. (%)	7153	58 (0.8)
Maternal illnesses and conditions		
Prepregnancy BMI, mean (SD) [range]	6516	22.9 (3.8) [12.5-51.2]
Age at delivery, mean (SD) [range], y	7486	28.5 (4.8) [15-44]
Ever had diabetes, No. (%)	6913	71 (1.0)
Gestational diabetes, No. (%)	7412	68 (0.9)
Ever had hypertension, No. (%)	6806	984 (14.5)
Gestational hypertension, No. (%)	7252	1058 (14.6)
Preeclampsia, No. (%)	7412	160 (2.2)
Any vaginal bleeding in pregnancy, No. (%)	6759	1198 (17.7)
Any infection in pregnancy, No. (%)	6683	1476 (22.1)
Ever had rheumatism, No. (%)	6817	295 (4.3)
Ever had psoriasis, No. (%)	6793	256 (3.8)
Ever had severe depression, No. (%)	6847	539 (7.9)
Stressful life event score, mean (SD) [range]		
First trimester	6841	3.6 (2.6) [0-18]
Third trimester	6695	3.5 (2.4) [0-26]
Pregnancy blood levels, mean (SD), [range]		
Vitamin D, pg/mL	4793	67.6 (32.1) [8.6-248.1]
Selenium, μg/L	2625	112.4 (24.0) [17.0-319.4]
Hemoglobin, g/dL	2535	2.1 (1.1) [0.3-11.5]
Cadmium, μg/L	2624	0.5 (0.6) [0.1-4.6]
Lead, μg/dL	2624	3.6 (1.5) [0.2-19.1]
Cesarean delivery, No. (%)	6660	674 (10.1)
Low birth weight (<2500 g), No. (%)	7387	328 (4.4)
Preterm delivery (<37 wk), No. (%)	7486	399 (5.3)
Hypoxia, No. (%)	4405	1301 (29.5)
Low Apgar score, No. (%)		
At 1 min	4387	539 (12.3)
At 5 min	4383	49 (1.1)
Breastfeeding at 1 mo, No. (%)	6490	5053 (77.9)

Abbreviation: BMI, body mass index (calculated as weight in kilograms divided by height in meters squared).

SI conversion factors: To convert cadmium to nanomoles per liter, multiply by 8.896; hemoglobin to grams per liter, multiply by 10.0; lead to micromoles per liter, multiply by 0.0483; selenium to micromoles per liter, multiply by 0.0127; vitamin D to picomoles per liter, multiply by 2.496.

**Table 2.** Associations of Maternal PRSs for ADHD, ASD, and Schizophrenia With Lifestyle-Related Prenatal Exposures

Variable	ADHD PRS		ASD PRS		SCZ PRS	
	OR or β Coefficient (95% CI)^a^	*P* Value	OR or β Coefficient (95% CI)^a^	*P* Value	OR or β Coefficient (95% CI)^a^	*P* Value
Smoking, OR						
During first trimester	1.25 (1.17 to 1.33)	1 × 10^−11^	1.04 (0.98 to 1.11)	.18	1.14 (1.04 to 1.18)	.001
During third trimester	1.27 (1.18 to 1.36)	6 × 10^−11^	1.06 (0.99 to 1.14)	.09	1.06 (0.99 to 1.14)	.08
Alcohol consumption, OR						
During first trimester	0.99 (0.92 to 1.06)	.76	1.00 (0.94 to 1.08)	.91	1.01 (0.94 to 1.08)	.85
During third trimester	0.91 (0.84 to 0.98)	.02	1.05 (0.97 to 1.13)	.22	1.05 (0.97 to 1.13)	.26
Binge drinking during first trimester	1.11 (1.03 to 1.18)	.004	1.05 (0.98 to 1.13)	.15	1.08 (1.01 to 1.16)	.02
Taking supplements during pregnancy, OR						
Iron	0.97 (0.91 to 1.03)	.31	1.00 (0.94 to 1.06)	.92	1.09 (1.02 to 1.16)	.01
Zinc	0.91 (0.74 to 1.13)	.40	0.98 (0.79 to 1.20)	.82	1.22 (0.99 to 1.50)	.06
Folic acid	0.96 (0.89 to 1.05)	.36	1.06 (0.98 to 1.15)	.17	1.11 (1.03 to 1.21)	.01
Vitamins	0.94 (0.88 to 1.01)	.09	0.99 (0.92 to 1.06)	.73	1.10 (1.03 to 1.18)	.005
Use of acetaminophen, OR						
In early pregnancy	1.09 (1.02 to 1.17)	.01	1.00 (0.94 to 1.08)	.93	0.97 (0.90 to 1.03)	.32
In late pregnancy	1.11 (1.04 to 1.18)	.002	0.99 (0.93 to 1.05)	.68	1.02 (0.95 to 1.08)	.62
Use of antidepressants, OR	1.06 (0.82 to 1.38)	.64	1.05 (0.81 to 1.36)	.70	1.02 (0.78 to 1.32)	.90
Prepregnancy BMI, β coefficient	0.27 (0.18 to 0.36)	8 × 10^−9^	−0.02 (−0.11 to 0.07)	.70	−0.16 (−0.26 to −0.07)	.001
Age at delivery, β coefficient	−0.42 (−0.53 to −0.31)	2 × 10^−14^	0.09 (−0.01 to 0.20)	.095	0.05 (−0.06 to 0.16)	.35

Abbreviations: ADHD, attention-deficit/hyperactivity disorder; ASD, autism spectrum disorder; BMI, body mass index (calculated as weight in kilograms divided by height in meters squared); OR, odds ratio; PRS, polygenic risk score; SCZ, schizophrenia.

^a^ Odds ratios were calculated for binary outcomes; β coefficients, for continuous outcomes.

#### Maternal Illnesses and Conditions

We found evidence of associations between all 3 maternal PRSs and a higher risk of experiencing severe depression (OR range, 1.12 [95% CI, 1.02-1.23] to 1.21 [95% CI, 1.11-1.33]) and an increased stressful life events score in the first trimester of pregnancy (β coefficient range, 0.11 [95% CI, 0.05-0.17] to 0.13 [95% CI, 0.07-0.19]) (Table 3). In addition, ADHD PRS showed evidence of an association with infection during pregnancy (OR, 1.11; 95% CI, 1.04-1.18). Little evidence was found for associations with other illnesses or conditions.

**Table 3.** Associations of Maternal PRSs for ADHD, ASD, and Schizophrenia With Maternal Illnesses and Conditions

Variable	ADHD PRS		ASD PRS		SCZ PRS	
	OR or β Coefficient (95% CI)^a^	*P* Value	OR or β Coefficient (95% CI)^a^	*P* Value	OR or β Coefficient (95% CI)^a^	*P* Value
Ever had diabetes, OR	1.30 (1.03 to 1.65)	.03	1.13 (0.89 to 1.43)	.31	1.01 (0.79 to 1.28)	.97
Gestational diabetes, OR	1.32 (1.03 to 1.67)	.03	1.04 (0.82 to 1.32)	.75	0.89 (0.70 to 1.13)	.33
Ever had hypertension, OR	1.06 (0.98 to 1.14)	.13	1.01 (0.94 to 1.09)	.74	1.07 (0.99 to 1.15)	.08
Gestational hypertension, OR	0.96 (0.90 to 1.03)	.27	0.99 (0.92 to 1.06)	.746	1.05 (0.97 to 1.12)	.21
Preeclampsia, OR	1.09 (0.93 to 1.28)	.30	1.18 (1.01 to 1.39)	.04	1.08 (0.92 to 1.26)	.37
Vaginal bleeding in pregnancy, OR	1.03 (0.96 to 1.10)	.47	1.01 (0.94 to 1.08)	.80	1.08 (1.00 to 1.15)	.04
Any infection in pregnancy, OR	1.11 (1.04 to 1.18)	.002	1.06 (0.99 to 1.13)	.09	1.03 (0.97 to 1.10)	.33
Ever had rheumatism, OR	1.05 (0.93 to 1.19)	.40	1.16 (1.03 to 1.31)	.02	1.03 (0.91 to 1.16)	.63
Ever had psoriasis, OR	1.00 (0.88 to 1.13)	.97	1.04 (0.92 to 1.18)	.53	1.04 (0.91 to 1.18)	.55
Ever had severe depression, OR	1.21 (1.11 to 1.33)	4 × 10^−5^	1.12 (1.02 to 1.23)	.01	1.21 (1.11 to 1.33)	5 × 10^−5^
Stressful life event score, β coefficient						
First trimester	0.13 (0.07 to 0.19)	4 × 10^−5^	0.11 (0.05 to 0.17)	3 × 10^−4^	0.12 (0.06 to 0.18)	2 × 10^−4^
Third trimester	0.08 (0.02 to 0.14)	.008	0.09 (0.004 to 0.03)	.004	0.05 (−0.01 to 0.11)	.09

Abbreviations: ADHD, attention-deficit/hyperactivity disorder; ASD, autism spectrum disorder; OR, odds ratio; PRS, polygenic risk score; SCZ, schizophrenia.

^a^ Odds ratios were calculated for binary outcomes; β coefficients, for continuous outcomes.

#### Biomarkers of Nutritional Status and Toxin Exposure in Pregnancy

Maternal ADHD PRS showed evidence of association with lower selenium levels (β coefficient, −0.01; 95% CI, −0.02 to 0.005) and higher cadmium levels (β coefficient, 0.07; 95% CI, 0.05-0.0009). In addition, ADHD PRS showed evidence of associations with lower mercury levels (β coefficient, −0.06; 95% CI, −0.11 to −0.02), but little evidence of association with vitamin D or lead (Table 4). Little evidence was found for associations between maternal ASD PRS and SCZ PRS and any of the biomarkers.

**Table 4.** Associations of Maternal PRSs for ADHD, ASD, and Schizophrenia With Maternal Blood Biomarkers of Nutritional Status and Toxins in Pregnancy

Biomarker	ADHD PRS		ASD PRS		SCZ PRS	
	β Coefficient (95% CI)	*P* Value	β Coefficient (95% CI)	*P* Value	β Coefficient (95% CI)	*P* Value
Vitamin D^a^	0.01 (−0.003 to 0.02)	.99	−0.01 (−0.02 to 0.01)	.24	0.01 (−0.003 to 0.02)	.12
Selenium	−0.01 (−0.02 to 0.00)	.001	0.001 (−0.01 to 0.01)	.84	0.004 (−0.004 to 0.01)	.30
Mercury	−0.06 (−0.11 to −0.02)	.003	0.02 (−0.02 to 0.06)	.42	0.03 (−0.02 to 0.07)	.24
Cadmium	0.07 (0.05 to 0.09)	1 × 10^−9^	0.01 (−0.01 to 0.03	.52	0.03 (0.004 to 0.05)	.02
Lead	−0.03 (−0.08 to 0.03)	.36	0.03 (−0.03 to 0.08)	.36	−0.01 (−0.07 to 0.04)	.63

Abbreviations: ADHD, attention-deficit/hyperactivity disorder; ASD, autism spectrum disorder; PRS, polygenic risk score; SCZ, schizophrenia.

^a^ Indicates log transformed.

#### Perinatal Factors and Conditions

We found little evidence of an association between maternal neurodevelopmental risk scores and birth events, including low birth weight, preterm delivery, cesarean delivery, and hypoxia (Table 5). Furthermore, there was no strong evidence of associations between these outcomes and child neurodevelopmental PRSs (Table 5).

**Table 5.** Associations of Maternal and Child PRSs for ADHD, ASD, and Schizophrenia With Perinatal Exposures and Conditions

Exposure	No. of Participants			ADHD PRS		ASD PRS		SCZ PRS	
	No	Yes	Total	OR (95% CI)	*P* Value	OR (95% CI)	*P* Value	OR (95% CI)	*P* Value
**Associations With Maternal PRSs**									
Cesarean delivery	5986	674	6660	0.99 (0.91-1.08)	.80	1.03 (0.95-1.12)	.47	0.95 (0.87-1.03)	.20
Low birth weight, <2500 g	7059	328	7387	1.08 (0.97-1.21)	.18	1.00 (0.90-1.13)	.94	1.02 (0.62-1.68)	.94
Preterm delivery, <37 wk	7087	399	7486	0.99 (0.89-1.10)	.85	0.96 (0.87-1.07)	.49	0.96 (0.87-1.07)	.46
Hypoxia	3104	1301	4405	1.05 (0.97-1.14)	.20	1.04 (0.97-1.13)	.28	1.02 (0.95-1.11)	.56
Low Apgar score									
At 1 min	3848	539	4387	1.11 (1.01-1.23)	.03	1.02 (0.93-1.13)	.65	1.00 (0.91-1.11)	.95
At 5 min	4334	49	4383	1.05 (0.79-1.39)	.74	1.04 (0.79-1.38)	.78	0.77 (0.57-1.03)	.07
Breastfeeding at 1 mo	1437	5053	6490	0.88 (0.78-0.99)	.04	1.05 (0.92-1.19)	.47	1.02 (0.90-1.16)	.75
**Associations With Child PRSs**									
Cesarean delivery	6126	686	6812	0.98 (0.90-1.07)	.62	0.97 (0.89-1.05)	.45	1.00 (0.92-1.08)	.92
Low birth weight, 2500 g	7118	298	7416	1.06 (0.94-1.19)	.36	0.97 (0.86-1.09)	.63	0.96 (0.85-1.09)	.54
Preterm delivery, <37 wk	7134	374	7508	1.03 (0.92-1.15)	.62	0.94 (0.85-1.05)	.30	0.95 (0.85-1.06)	.37
Hypoxia	3147	1256	4403	0.97 (0.90-1.05)	.52	0.99 (0.91-1.07)	.74	0.98 (0.91-1.06)	.66
Low Apgar score									
At 1 min	3886	511	4397	1.01 (1.00-1.01)	.27	1.00 (0.99-1.01)	.69	1.00 (0.99-1.01)	.45
At 5 min	4348	47	4395	1.00 (1.00-1.01)	.13	1.00 (1.00-1.00)	.44	1.00 (1.00-1.00)	.56
Breastfeeding at 1 mo	1351	5701	7052	0.89 (0.78-1.02)	.10	1.06 (0.93-1.22)	.39	1.04 (0.91-1.19)	.60

Abbreviations: ADHD, attention-deficit/hyperactivity disorder; ASD, autism spectrum disorder; OR, odds ratio; PRS, polygenic risk score; SCZ, schizophrenia.

### Sensitivity Analyses

Sensitivity analysis revealed consistent results across different *P* value thresholds for all 3 PRSs, as shown in eFigures 1 to 6 in the Supplement. To account for attrition, we performed inverse probability weighting on availability of genetic data and reran our main analysis with the derived weights, which revealed a similar pattern of results (eTables 6 and 7 in the Supplement).

In addition, we evaluated the observational association of the investigated early-life exposures with ADHD and ASD symptoms in children in the ALSPAC population (eMethods and eTable 8 in the Supplement). We found evidence of an association of smoking (relative risk [RR], 1.70; 95% CI, 1.37-2.10) and use of acetaminophen (RR, 1.45; 95% CI, 1.18-1.78) during pregnancy, ever having depression (RR, 1.64; 95% CI, 1.20-2.25), and an increased stressful life events score (RR, 1.15; 95% CI, 1.10-1.20) with an increased risk of ADHD, as described previously for this cohort.^14,31^

## Discussion

The present study examines the association between neurodevelopmental disorder PRSs and early-life exposures associated with those disorders. We found that maternal risk alleles for neurodevelopmental disorders, primarily ADHD, were associated with some prenatal factors. These findings are in line with those of studies showing substantial associations between genetic factors and environmental experiences^56^ and highlight the need to account for genetic confounding in studies of pregnancy-related exposures and neurodevelopmental disorders.

Only maternal stressful life events during pregnancy and lifetime depression showed consistent associations with PRS for all 3 neurodevelopmental disorders. These associations are in line with previous findings suggesting that some stressful life events are heritable^57^ and that stressful life events, depression, and neurodevelopmental disorders share genetic risk factors.^58,59^ Apart from horizontal pleiotropy, a potential explanation for the PRS associations with stressful life events is that maternal traits of neurodevelopmental disorders could be associated with stressful situations, making mothers at high genetic risk for ADHD more likely to encounter stressful events.

Maternal ADHD PRS showed associations with a variety of prenatal factors. Consistent with previous studies, we found that genetic liability for ADHD was associated with higher BMI, younger maternal age at delivery, and smoking.^35^ To our knowledge, this study was the first to report that genetic liability to ADHD may also be associated with infections, acetaminophen use, and blood levels of toxins in pregnant mothers. Acetaminophen exposure in pregnancy is of considerable current interest because studies have raised concern about its safety owing to the observed association with offspring ADHD.^60^ Our results suggest that mothers with higher ADHD PRS may also be more likely to use acetaminophen in pregnancy. Whether the association between ADHD and acetaminophen use holds after adjusting for shared genetic factors and represents a causal relationship should be assessed in a causally informative mendelian randomization framework and with other genetically informative designs that test and account for horizontal pleiotropy.^61,62^

The association between ADHD PRS and lower biomarker levels of selenium and mercury may seem counterintuitive. However, because fish is a major source of these heavy metals in the general population and ADHD is associated with low fish consumption,^63^ a possible explanation could be that mothers with higher ADHD PRS consumed less fish.

In contrast to ADHD PRS, we found little evidence of associations between ASD PRS or SCZ PRS and early-life factors (except for SCZ PRS with lower BMI and more smoking, consistent with previous studies). It is possible that ADHD risk alleles have especially widespread pleiotropic or causal effects on lifestyle and risk-taking behavior during pregnancy. In addition, the differential pattern of associations could be driven in part by genetic overlap between the 3 disorders and educational attainment. Although ADHD has shown genetic overlap with lower educational attainment,^35^ genetic overlap with higher educational attainment has been found for ASD and less strongly for schizophrenia.^59,64^ Educational attainment has been shown to be associated with health-conscious behavior^65^ and pregnancy-related exposures.^66^ The differential associations with educational attainment might also explain why many of the coefficients for ASD PRS and SCZ PRS were in the opposite direction from ADHD PRS (eg, age at childbirth, selenium levels, folic acid intake, and breastfeeding). Future studies could use a multipolygenic score model to assess the extent to which associations between neurodevelopmental disorder risk alleles and prenatal factors may be explained by education-associated alleles.^67^

Our results have implications for supporting pregnant women with neurodevelopmental problems such as ADHD, given that maternal neurodevelopmental risk alleles were associated with exposure to adverse conditions (eg, stress) and risky behaviors (eg, smoking) in pregnancy. The associations between ADHD PRS and prenatal exposures were generally of small magnitude, and additional types of investigations are needed to assess whether the associations are of clinical importance. Nevertheless, our findings add to the increasing evidence that the observational associations between many prenatal factors and neurodevelopmental disorders in offspring may be at least partially genetically confounded. However, the presence of genetic confounding does not exclude a causal effect. Genetic effects may even be mediated through an environmental exposure. For example, in the present study, we found that maternal ADHD PRS was associated with higher levels of the heavy metal cadmium, for which smoking is a common source.^68^ Although maternal genetic liability for ADHD predisposes mothers to smoke during pregnancy, prenatal cadmium exposure due to smoking could still be associated with an increased risk for ADHD in offspring. However, this increased risk has been shown to be unlikely by triangulation of evidence from studies comparing smoking during pregnancy between mothers and fathers and between genetically related and unrelated mothers (in vitro fertilization cohort).^34,44^ Disentangling complex questions of whether prenatal factors causally influence risk of neurodevelopmental disorders in offspring will likely require such triangulation of evidence from multiple approaches that rely on different assumptions and have unrelated sources of potential biases,^69,70^ including genetically informed methods.^62^

### Limitations

ALSPAC did not assess diagnosis of maternal ADHD or ASD, and only 7 mothers reported a diagnosis of SCZ; therefore, we were not able to evaluate the estimation of our generated PRSs or test for the association of observed maternal neurodevelopmental traits with early-life exposures in ALSPAC. Nevertheless, previous studies of children in the ALSPAC population have found robust associations between ADHD PRS and ADHD symptoms,^71^ associations between ASD PRS and ASD symptoms,^71^ and associations between SCZ PRS and negative symptoms of SCZ.^39^

Polygenic risk scores have been demonstrated to be useful instruments for polygenic traits commonly used to investigate the genetic architecture of many disorders.^40^ However, PRSs currently explain only a small amount of variance in heritability of neurodevelopmental disorders.^35,36,72^ Our power to detect associations with exposures may have been low for ASD because its SNP heritability is lower than that for ADHD and SCZ.^73^

Given that ADHD PRS (as well as SCZ PRS) is associated with study attrition,^74^ we performed inverse probability weighting sensitivity analyses accounting for the probability of having genetic data available. The weighted analyses showed similar results. Nevertheless, given that not all selection-associated factors could be included in the weighting, bias may remain. Furthermore, sample sizes were small for some early-life exposures, and the nested studies for heavy metal detection in pregnancy might be limited by selection bias. Although we have accounted for multiple testing, we must acknowledge that some of our findings might still occur by chance. Our study relied on self-reported smoking and alcohol consumption. These exposures may be underreported, especially during pregnancy, when smoking and alcohol consumption is considered socially undesirable, but results were consistent when comparing self-reported smoking and alcohol consumption before pregnancy (eTable 9 in the Supplement). We also did not have information about maternal use of medication to treat symptoms of ADHD, ASD, or schizophrenia and therefore cannot exclude the possibility that potential intake of medication for maternal mental health has led to spurious associations. However, excluding all mothers who reported they had taken medication for neuropsychiatric problems (ie, depression, anxiety, migraine, or sleeping problems) did not change our results (eTable 10 in the Supplement). We encourage replication of our findings in well-powered data sets.

## Conclusions

Our findings suggest that several early-life factors linked to neurodevelopmental disorders are associated with maternal genetic liabilities to these disorders, primarily ADHD. Therefore, to draw conclusions about causality, future studies need to account for potential genetic confounding and triangulate evidence from different causally informative approaches. In addition, mothers with high genetic liability to ADHD may be at increased risk for many adverse pregnancy factors.

## Notes

Supplement. **eMethods.** Calculation of Polygenic Risk Scores and Analyses **eTable 1.** Number of SNPs Used to Calculate the Polygenic Risk Scores (PRS) for ADHD, ASD, and Schizophrenia **eTable 2.** Correlation (*r*) of Polygenic Risk Scores (PRS) for ADHD, ASD, and Schizophrenia **eTable 3.** Principal Component Analysis of Outcomes to Determine the Number of Independent Tests **eTable 4.** Comparison of Study Sample With and Without Available Genetics Data **eTable 5.** Association of Maternal Polygenic Risk Scores (PRS) for ADHD, ASD, and Schizophrenia (*P* < .05) With Maternal Age at Delivery **eTable 6.** Association of Maternal Polygenic Risk Scores (PRS) (*P* < .05) With Prenatal Exposures Linked to Offspring Neurodevelopmental Disorders **eTable 7.** Association of Maternal Polygenic Risk Scores (PRS) (*P* < .05) With Perinatal Exposures and Conditions Linked to Offspring Neurodevelopmental Disorders **eTable 8.** Association of Early Life Exposure With Observed ADHD and ASD Symptoms in ALSPAC **eTable 9.** Association of Maternal Polygenic Risk Scores (*P* < .05) With Maternal Alcohol Consumption, Smoking Behavior, and Stressful Life Events at Different Time Intervals During and Before Pregnancy **eTable 10.** Association of Maternal Polygenic Risk Scores (PRS) (*P* < .05) With Prenatal Exposures Linked to Offspring Neurodevelopmental Disorders, Excluding Mothers Who Have Reported Taking Medication for Anxiety, Depression, Migraine, Sleeping Problem, or Other **eFigure 1.** Association of Maternal Polygenic Risk Scores for ADHD at Different *P* Value Thresholds With Prenatal and Perinatal Exposures Linked to Offspring Neurodevelopmental Disorders With Continuous Outcomes **eFigure 2.** Association of Maternal Polygenic Risk Scores for ADHD at Different *P* Value Thresholds With Prenatal and Perinatal Exposures Linked to Offspring Neurodevelopmental Disorders With Binary Outcomes **eFigure 3.** Association of Maternal Polygenic Risk Scores for Autism at Different *P* Value Thresholds With Prenatal and Perinatal Exposures Linked to Offspring Neurodevelopmental Disorders With Continuous Outcomes **eFigure 4.** Association of Maternal Polygenic Risk Scores for Autism at Different *P* Value Thresholds With Prenatal and Perinatal Exposures Linked to Offspring Neurodevelopmental Disorders With Binary Outcomes **eFigure 5.** Association of Maternal Polygenic Risk Scores for Schizophrenia at Different *P* Value Thresholds With Prenatal and Perinatal Exposures Linked to Offspring Neurodevelopmental Disorders With Continuous Outcomes **eFigure 6.** Association of Maternal Polygenic Risk Scores for Schizophrenia at Different *P* Value Thresholds With Prenatal and Perinatal Exposures Linked to Offspring Neurodevelopmental Disorders With Binary Outcomes
