# Supplementary material for: Association of Maternal Neurodevelopmental Risk Alleles With Early-Life Exposures
Source: JAMA Psychiatry. 2019 May 1;76(8):834–42. doi: 10.1001/jamapsychiatry.2019.0774 (PMC6495368; doi:10.1001/jamapsychiatry.2019.0774)
Supplement: Supplement. — eMethods. Calculation of Polygenic Risk Scores and Analyses eTable 1. Number of SNPs Used to Calculate the Polygenic Risk Scores (PRS) for ADHD, ASD, and Schizophrenia eTable 2. Correlation (r) of Polygenic Risk Scores (PRS) for ADHD, ASD, and Schizophrenia eTable 3. Principal Component Analysis of Outcomes to Determine the Number of Independent Tests eTable 4. Comparison of Study Sample With and Without Available Genetics Data eTable 5. Association of Maternal Polygenic Risk Scores (PRS) for ADHD, ASD, and Schizophrenia (P < .05) With Maternal Age at Delivery eTable 6. Association of Maternal Polygenic Risk Scores (PRS) (P < .05) With Prenatal Exposures Linked to Offspring Neurodevelopmental Disorders eTable 7. Association of Maternal Polygenic Risk Scores (PRS) (P < .05) With Perinatal Exposures and Conditions Linked to Offspring Neurodevelopmental Disorders eTable 8. Association of Early Life Exposure With Observed ADHD and ASD Symptoms in ALSPAC eTable 9. Association of Maternal Polygenic Risk Scores (P < .05) With Maternal Alcohol Consumption, Smoking Behavior, and Stressful Life Events at Different Time Intervals During and Before Pregnancy eTable 10. Association of Maternal Polygenic Risk Scores (PRS) (P < .05) With Prenatal Exposures Linked to Offspring Neurodevelopmental Disorders, Excluding Mothers Who Have Reported Taking Medication for Anxiety, Depression, Migraine, Sleeping Problem, or Other eFigure 1. Association of Maternal Polygenic Risk Scores for ADHD at Different P Value Thresholds With Prenatal and Perinatal Exposures Linked to Offspring Neurodevelopmental Disorders With Continuous Outcomes eFigure 2. Association of Maternal Polygenic Risk Scores for ADHD at Different P Value Thresholds With Prenatal and Perinatal Exposures Linked to Offspring Neurodevelopmental Disorders With Binary Outcomes eFigure 3. Association of Maternal Polygenic Risk Scores for Autism at Different P Value Thresholds With Prenatal and Perinatal Exposures Linked to Offsprin [file jamapsychiatry-76-834-s001.pdf]

Supplementary Online Content

Leppert B, Havdahl A, Riglin L, et al. Association of maternal neurodevelopmental risk alleles with early-life exposures. *JAMA Psychiatry*. Published online May 1, 2019. doi:10.1001/jamapsychiatry.2019.0774

eMethods. Calculation of Polygenic Risk Scores and Analyses

eTable 1. Number of SNPs Used to Calculate the Polygenic Risk Scores (PRS) for ADHD, ASD, and Schizophrenia

eTable 2. Correlation (r) of Polygenic Risk Scores (PRS) for ADHD, ASD, and Schizophrenia

eTable 3. Principal Component Analysis of Outcomes to Determine the Number of Independent Tests

eTable 4. Comparison of Study Sample With and Without Available Genetics Data

eTable 5. Association of Maternal Polygenic Risk Scores (PRS) for ADHD, ASD, and Schizophrenia (P < .05) With Maternal Age at Delivery

eTable 6. Association of Maternal Polygenic Risk Scores (PRS) (P < .05) With Prenatal Exposures Linked to Offspring Neurodevelopmental Disorders

eTable 7. Association of Maternal Polygenic Risk Scores (PRS) (P < .05) With Perinatal Exposures and Conditions Linked to Offspring Neurodevelopmental Disorders

eTable 8. Association of Early Life Exposure With Observed ADHD and ASD Symptoms in ALSPAC

eTable 9. Association of Maternal Polygenic Risk Scores (P < .05) With Maternal Alcohol Consumption, Smoking Behavior, and Stressful Life Events at Different Time Intervals During and Before Pregnancy

eTable 10. Association of Maternal Polygenic Risk Scores (PRS) (P < .05) With Prenatal Exposures Linked to Offspring Neurodevelopmental Disorders, Excluding Mothers Who Have Reported Taking Medication for Anxiety, Depression, Migraine, Sleeping Problem, or Other

eFigure 1. Association of Maternal Polygenic Risk Scores for ADHD at Different P Value Thresholds With Prenatal and Perinatal Exposures Linked to Offspring Neurodevelopmental Disorders

eFigure 2. Association of Maternal Polygenic Risk Scores for ADHD at Different P Value Thresholds With Prenatal and Perinatal Exposures Linked to Offspring Neurodevelopmental Disorders

© 2019 Leppert B et al. *JAMA Psychiatry.*

eFigure 3. Association of Maternal Polygenic Risk Scores for Autism at Different P Value Thresholds With Prenatal and Perinatal Exposures Linked to Offspring Neurodevelopmental Disorders

eFigure 4. Association of Maternal Polygenic Risk Scores for Autism at Different P Value Thresholds With Prenatal and Perinatal Exposures Linked to Offspring Neurodevelopmental Disorders

eFigure 5. Association of Maternal Polygenic Risk Scores for Schizophrenia at Different P Value Thresholds With Prenatal and Perinatal Exposures Linked to Offspring Neurodevelopmental Disorders

eFigure 6. Association of Maternal Polygenic Risk Scores for Schizophrenia at Different P Value Thresholds With Prenatal and Perinatal Exposures Linked to Offspring Neurodevelopmental Disorders

This supplementary material has been provided by the authors to give readers additional information about their work.

© 2019 Leppert B et al. *JAMA Psychiatry.*

eMethods. Calculation of Polygenic Risk Scores and Analyses

Polygenic risk score

PRSs were calculated using PRSice v1.25 and PLINK.v.1.9 as the weighted, mean number of disorder risk alleles in approximate linkage equilibrium (R^2<0.1 within 1000kb distance), as has been described previously.[45] Risk alleles for ADHD and ASD were defined as those identified in the most recent combined Psychiatric Genomics Consortium (PGC) and iPSYCH analysis of case-control GWAS[35,36], at a threshold of p<0.05 to maximize phenotypic variance. In brief, the meta-analysis for ADHD included 20,183 ADHD cases and 35,191 controls and meta-analysis for autism included 18,381 autism cases and 27,969 controls of European ancestry. Risk alleles for schizophrenia were identified by the PGC meta-analysis[37] including 34,241 schizophrenia cases and 45,604 controls at a threshold of p<0.05. All summary statistics were subject to standard quality control including filtering for minor allele frequency (MAF>0.1) and imputation quality (INFO>0.8)[46], according to an in-house algorithm developed by Richard Anney (https://github.com/ricanney). The MHC region on chromosome 6 (26-33Mb) was excluded due to its complex linkage disequilibrium (LD) structure. PRS were standardized using z-score transformation. Correlations between PRS (p<0.05) were as follows: PRS_{ADHD}-PRS_{ASD} r=0.22, PRS_{ADHD}-PRS_{SCZ} r=0.05, PRS_{ASD}-PRS_{SCZ} r=0.05. For sensitivity analysis PRS were derived using various p-value thresholds (p<0.5, p<0.1, p<0.01, p<0.005, p<0.001). The number of SNPs included for each threshold and the corresponding correlations between PRS are shown in Supplementary Table S1 and S2, respectively.

© 2019 Leppert B et al. *JAMA Psychiatry.*

Principal Component Analysis (PCA)

To correct for multiple testing of 32 early-life exposures, the number of independent tests was determined based on the number of PCs that explained 80% of covariance between the early-life exposures in an unrotated principal component analysis (PCA) using the `pca` function in STATA 13 (see Table S3). We conclude that 21 independent tests were performed leading to a multiple testing corrected p-value of $p<0.0024$ (0.05/21), which is slightly less conservative than the Bonferroni corrected p-value of $p<0.0016$ (0.05/32), assuming all tests to be independent.

Inverse probability weighting

PRS for ADHD and schizophrenia have been shown to be highly associated with attrition in the ALSPAC core study[52] and a comparison between the study sample with (n=7,486) and without (n=6,307) genetic data available suggests that they differ in many of the analysed factors, especially those that relate to socio-economic status (Table S2). As sensitivity analysis, we performed an inverse probability weighting on missing maternal genetic data. Weights were derived from a logistic regression model for missingness including variables with <1% missing data (child sex, birthweight and maternal age at delivery) in the ALSPAC core study. Missing data for maternal age at delivery was imputed as the mean value. Weights ranged from 1.36 to 3.03.

Observational associations

ADHD was defined as score of more or equal to 7 on the 5 items hyperactivity subscale of the Strength and Difficulties Questionnaire (SDQ) at age 7. ASD was defined as either a) being diagnosed with Pervasive Developmental Disorder using questions from the DAWBA questionnaire at 91months or b) the mother's answer to

© 2019 Leppert B et al. *JAMA Psychiatry.*

the question “Have you ever been told that your child has autism, Asperger’s syndrome or autistic spectrum disorder?” at age 9 or c) a diagnosis of autism spectrum disorder using the ICD-10 criteria from a review of all children given a statement for special educational provision in the Avon area [1].

Risk ratios (RR) for the investigated early life exposures were assessed using general linear models (glm) with logit-link and Poisson distribution, adjusted for sex and age at ADHD/ASD assessment.

1 *Williams E, Thomas K, Sidebotham H, Emond A.* Prevalence and characteristics of autistic spectrum disorders in the ALSPAC cohort. *Dev Med Child Neurol. 2008 Sep; 50(9):672-7.*

eTables

eTable 1. Number of SNPs Used to Calculate the Polygenic Risk Scores (PRS) for ADHD, ASD, and Schizophrenia

	Number of SNPs used to calculate:		
p-value threshold	PRS_{ADHD}	PRS_{ASD}	PRS_{SCZ}
0.5	76371	102902	79956
0.1	29240	37678	35564
0.05	18475	23085	24367
0.01	6156	7040	10165
0.005	3800	4135	7014
0.001	1303	1259	3075
5x10-8	16	12	108

© 2019 Leppert B et al. *JAMA Psychiatry.*

eTable 2. Correlation (*r*) of Polygenic Risk Scores (PRS) for ADHD, ASD, and Schizophrenia

P value threshold	**.50**	**.10**	**.05**	**.01**	**.005**	**.001**
**a) PRS_{ADHD}**						
**0.5**	1					
**0.1**	0.90	1				
**0.05**	0.83	0.92	1			
**0.01**	0.64	0.71	0.77	1		
**0.005**	0.55	0.61	0.66	0.87	1	
**0.001**	0.39	0.43	0.47	0.61	0.71	1
**b) PRS_{ASD}**						
**0.5**	1					
**0.1**	0.89	1				
**0.05**	0.82	0.91	1			
**0.01**	0.82	0.91	1	1		
**0.005**	0.53	0.59	0.64	0.64	1	
**0.001**	0.36	0.40	0.44	0.44	0.66	1
**c) PRS_{SCZ}**						
**0.5**	1					
**0.1**	0.93	1				
**0.05**	0.88	0.94	1			
**0.01**	0.75	0.80	0.85	1		
**0.005**	0.69	0.74	0.78	0.92	1	
**0.001**	0.55	0.59	0.64	0.75	0.82	1
**d) PRS correlation across disorders***						
p-value threshold	**0.5**	**0.1**	**0.05**	**0.01**	**0.005**	**0.001**
**1) Correlation with PRS_{ADHD}**						
**PRS_{ASD}**	0.26	0.24	0.22	0.17	0.12	0.08
**PRS_{SCZ}**	0.05	0.05	0.05	0.05	0.05	0.04
**2) Correlation with PRS_{ASD}**						
**PRS_{SCZ}**	0.06	0.06	0.05	0.04	0.01	0.01

*with same p-value threshold

© 2019 Leppert B et al. *JAMA Psychiatry.*

eTable 3. Principal Component Analysis of Outcomes to Determine the Number of Independent Tests

Component	Eigenvalue	Difference	Proportion	Cumulative
Comp1	2.41	0.14	0.07	0.07
Comp2	2.27	0.35	0.07	0.14
Comp3	1.92	0.19	0.06	0.20
Comp4	1.73	0.06	0.05	0.25
Comp5	1.67	0.14	0.05	0.30
Comp6	1.53	0.21	0.05	0.35
Comp7	1.31	0.01	0.04	0.39
Comp8	1.30	0.12	0.04	0.43
Comp9	1.18	0.05	0.04	0.46
Comp10	1.13	0.01	0.03	0.50
Comp11	1.12	0.05	0.03	0.53
Comp12	1.07	0.04	0.03	0.57
Comp13	1.03	0.03	0.03	0.60
Comp14	1.00	0.03	0.03	0.63
Comp15	0.98	0.01	0.03	0.66
Comp16	0.96	0.05	0.03	0.69
Comp17	0.92	0.02	0.03	0.71
Comp18	0.90	0.06	0.03	0.74
Comp19	0.84	0.01	0.03	0.77
Comp20	0.83	0.07	0.03	0.79
Comp21	0.76	0.01	0.02	0.81
Comp22	0.75	0.05	0.02	0.84
Comp23	0.70	0.03	0.02	0.86
Comp24	0.67	0.02	0.02	0.88
Comp25	0.66	0.05	0.02	0.90
Comp26	0.61	0.01	0.02	0.92
Comp27	0.60	0.04	0.02	0.93
Comp28	0.56	0.07	0.02	0.95
Comp29	0.50	0.05	0.02	0.97
Comp30	0.45	0.05	0.01	0.98
Comp31	0.40	0.16	0.01	0.99
Comp32	0.24	0.24	0.01	1.00

(unrotated rho=1, observations n=615)

© 2019 Leppert B et al. *JAMA Psychiatry.*

eTable 4. Comparison of Study Sample With and Without Available Genetics Data

	With genetic data			Without genetic data			*t* Test
	Mean	*SD*	*N*	*Mean*	*SD*	*N*	
Age at delivery	28.5	0.06	7486	27.4	0.06	6307	<.001
Stressful life event score	3.5	0.03	6695	3.5	0.04	4974	.889
Vitamin D (25OHD) [nmol/l]	67.6	0.46	4793	65.7	0.6	3017	.014
BMI (pre-pregnancy)	22.9	0.05	6516	22.9	0.06	4867	.817
Selenium (Se) [µg/l]	112	0.47	2625	111	0.65	1454	.030
Mercury (Hg) [µg/l]	2.12	0.02	2535	2.01	0.03	1396	.002
Cadmium (Cd) [µg/l]	0.52	0.01	2624	0.63	0.02	1451	<.001
Lead (Pb) [µg/l]	3.65	0.03	2624	3.71	0.04	1450	.212
	N(Yes)	*N(No)*	*N(total)*	*N(Yes)*	*N(No)*	*N(total)*	χ^2 Test
Smoking	1706	5148	6854	1837	3456	5293	<.001
Alcohol consumption	4312	1558	5900	3173	1061	4234	.036
Binge drinking at 1st trimester	1165	5968	7133	1016	4641	5657	.015
Breast fed their child	5562	1344	6906	3827	1447	5274	<.001
Taking iron supplements	1447	5662	7139	1400	4317	5717	<0.001
Taking zinc supplements	91	7048	7139	72	5643	5715	.940
Taking folic acid supplements	643	6489	7132	505	5206	5711	.733
Taking vitamin supplements	1166	5956	7122	929	4763	5692	.939
Taking paracetamol	3838	3304	7142	3182	2548	5730	.042
Taking antidepressants	58	7095	7153	66	5649	5715	.047
Ever had diabetes	68	7351	7419	39	6117	6156	.063
Gestational Diabetes	35	6615	6650	21	5084	5105	.37
Ever had hypertension	984	5822	6806	799	4453	5252	.247
Gestational hypertension	1058	6201	7259	869	5157	6026	.802
Preeclampsia	160	7259	7149	130	6026	6156	.857
Vaginal bleeding	1198	5561	6759	881	4327	5208	.247
Any infection	1476	5207	6683	1169	3793	4962	.061
Ever had rheumatism	295	6522	6817	228	4991	5219	.912
Ever had psoriasis	256	6537	6793	158	5039	5197	.030
Ever had depression	539	6308	6847	566	4694	5260	<.001

© 2019 Leppert B et al. *JAMA Psychiatry.*

eTable 5. Association of Maternal Polygenic Risk Scores (PRS) for ADHD, ASD, and Schizophrenia (P < .05) With Maternal Age at Delivery

				PRS_{ADHD}				PRS_{ASD}				PRS_{SCZ}		
		N		*OR*	*95% CI*	*P value*		*OR*	*95% CI*	*P value*		*OR*	*95% CI*	*P value*
Maternal age														
22 years and younger		768		1.32	1.22, 1.43	1×10^{-11}		0.96	0.89, 1.04	.298		0.95	0.87, 1.03	.186
23 – 27 years		2381		1.12	1.06, 1.18	6×10^{-5}		0.95	0.90, 1.00	.052		0.94	0.89, 1.00	.035
33 – 37 years		1212		0.98	0.91, 1.05	.538		1.01	0.94, 1.07	.879		0.94	0.88, 1.01	.086
38 years and older		256		0.89	0.79, 1.02	.088		1.02	0.90, 1.16	.769		1.06	0.93, 1.20	.407

Estimates from multinomial logistic regression with reference group for maternal age being 28-32 years (N=2869)
ADHD attention deficit/hyperactivity disorder, *OR* odds ratio, *CI* confidence interval

© 2019 Leppert B et al. *JAMA Psychiatry.*

eTable 6. Association of Maternal Polygenic Risk Scores (PRS) (*P* < .05) With Prenatal Exposures Linked to Offspring Neurodevelopmental Disorders

		PRS_{ADHD}				PRS_{ASD}				PRS_{SCZ}		
		OR	*95% CI*	*P value*		*OR*	*95% CI*	*P value*		*OR*	*95% CI*	*P value*
Smoking		1.26	1.20, 1.32	3×10^{-22}		1.03	0.98, 1.08	.230		1.11	1.06, 1.16	1×10^{-5}
Alcohol consumption		1.00	0.92, 1.08	.913		1.02	0.94, 1.11	.604		1.04	0.95, 1.13	.413
Binge drinking during 1st trimester		1.10	1.05, 1.16	1×10^{-4}		1.06	1.00, 1.11	.035		1.09	1.04, 1.15	.001
Taking iron supplements		0.98	0.93, 1.03	.371		1.00	0.95, 1.05	.979		1.09	1.03, 1.14	.001
Taking zinc supplements		0.97	0.3, 1.13	.689		0.99	0.84, 1.15	.855		1.23	1.05, 1.44	.009
Taking folic acid supplements		0.98	0.92, 1.04	.526		1.06	0.99, 1.13	.087		1.11	1.04, 1.19	.001
Taking vitamins supplements		0.92	0.77, 1.10	.347		0.89	0.75, 1.06	.203		1.05	0.88, 1.25	.597
Taking paracetamol		1.09	1.04, 1.15	.001		1.00	0.95, 1.06	.891		0.97	0.92, 1.02	.256
Taking antidepressants		1.06	0.88, 1.27	.563		1.08	0.89, 1.30	.448		1.03	0.85, 1.24	.748
Ever had diabetes		1.30	1.09, 1.55	.004		1.05	0.87, 1.25	.615		0.89	0.74, 1.06	.191
Gestational diabetes		1.50	1.17, 1.92	.001		1.01	0.79, 1.30	.933		1.08	0.84, 1.38	.563
Ever had hypertension		1.06	1.00, 1.01	.046		1.01	0.96, 1.07	.604		1.07	1.01, 1.13	.023
Gestational hypertension		0.97	0.92, 1.02	.267		0.99	0.94, 1.05	.771		1.04	0.99, 1.10	.120
Preeclampsia		1.09	0.97, 1.22	.148		1.19	1.06, 1.34	.003		1.08	0.96, 1.21	.208
Vaginal bleeding		1.03	0.98, 1.08	.293		1.01	0.96, 1.06	.692		1.07	1.02, 1.13	.008
Any infection		1.12	1.07, 1.17	6×10^{-6}		1.06	1.01, 1.11	.017		1.04	0.99, 1.09	.163
Ever had rheumatism		1.04	0.85, 1.14	.351		1.10	1.01, 1.20	.033		1.03	0.94, 1.13	.510
Ever had psoriasis		0.99	0.90, 1.09	.809		1.02	0.93, 1.12	.639		1.04	0.94, 1.14	.440
Ever had severe depression		1.22	1.14, 1.31	1×10^{-8}		1.12	1.05, 1.20	.001		1.21	1.13, 1.30	6×10^{-8}

© 2019 Leppert B et al. *JAMA Psychiatry.*

	PRS_{ADHD}			PRS_{ASD}			PRS_{SCZ}		
	β	*95% CI*	*P value*	*β*	*95% CI*	*P value*	*β*	*95% CI*	*P value*
BMI (pre-pregnancy)	0.27	0.17, 0.36	1 × 10-8	-0.02	-0.11, 0.07	.691	-0.16	-0.25, -0.07	.001
Age at delivery	-0.43	-0.54, -0.33	3×10^{-15}	0.10	-0.01, 0.20	.084	0.04	-0.06, 0.15	.426
Stressful life event score in 1st trimester	0.08	0.03, 0.14	.005	0.08	0.02, 0.14	.005	0.05	-0.01, 0.11	.112
Vitamin D (OHD)*	0.00	-0.01, 0.01	.991	-0.01	-0.02, 0.01	.300	0.11	0.00, 0.03	.097
Selenium (Se)*	-0.01	-0.02, -0.01	7×10^{-4}	0.00	-0.01, 0.01	.929	0.00	0.00, 0.01	.367
Mercury (Hg)	-0.07	-0.11, -0.02	0.002	0.01	-0.03, 0.06	0.506	0.02	-0.02, 0.06	0.297
Cadmium (Cd)	0.07	0.05, 0.09	$7x10^{-10}$	0.01	-0.01, 0.03	0.455	0.03	0.00, 0.05	0.019
Lead (Pb)	-0.03	-0.08, 0.03	0.361	0.02	-0.03, 0.08	0.385	-0.01	-0.07, 0.04	0.642

ADHD attention deficit/hyperactivity disorder, β beta coefficients, *CI* confidence interval, * log transformed

Analysis uses IPW to account for study population attrition.

© 2019 Leppert B et al. *JAMA Psychiatry.*

eTable 7. Association of Maternal Polygenic Risk Scores (PRS) (*P* < .05) With Perinatal Exposures and Conditions Linked to Offspring Neurodevelopmental Disorders

		PRS_{ADHD}				PRS_{ASD}				PRS_{SCZ}	
		OR	*95% CI*	*P value*		*OR*	*95% CI*	*P value*		*OR*	*95% CI*	*P value*
C-Section		1.00	0.95, 1.06	.888		1.02	0.96, 1.08	.460		0.96	0.90, 1.01	.132
Low birthweight (<2500g)		0.88	0.60, 1.30	.527		0.99	0.68, 1.43	.947		1.02	0.72, 1.43	.916
Preterm delivery (<37weeks)		0.99	0.92, 1.07	.814		0.97	0.90, 1.04	.351		0.95	0.89, 1.03	.226
Hypoxia		1.05	0.99, 1.11	.083		1.03	0.98, 1.09	.229		1.02	0.96, 1.08	.561
Low Apgar at 1 minute		1.12	1.04, 1.20	.002		1.02	0.95, 1.09	.587		1.00	0.93, 1.08	.974
Low Apgar at 5 minutes		1.06	0.86, 1.30	.579		1.07	0.87, 1.31	.536		0.76	0.62, 0.94	.012
Breast fed their child		0.87	0.79, 0.96	.004		1.04	0.94, 1.11	.460		1.04	0.95, 1.15	.416

ADHD attention deficit/hyperactivity disorder, *OR* odds ratio, *CI* confidence interval

Analysis uses IPW to account for study population attrition.

eTable 8. Association of Early Life Exposure With Observed ADHD and ASD Symptoms in ALSPAC

	ADHD					*ASD*				
	RR	*95% CI*	*P value*	N_{yes}	N_{No}	*RR*	*95% CI*	*P value*	N_{yes}	N_{No}
Smoking	1.70	1.37,2.10	1 × 10-6	1,666	5,923	0.77	0.45,1.30	.327	1,657	6,100
Alcohol consumption	1.42	1.09,1.85	.009	4,610	1,661	1.21	0.72,2.04	.467	4,721	1,699
Binge drinking*	1.35	1.05,1.72	.017	1,198	6,471	0.80	0.44,1.45	.465	1,202	6,669
Taking iron supplements	1.01	0.79,1.28	.958	1,576	6,099	1.28	0.81,2.03	.295	1,616	6,275
Taking zinc supplements	0.85	0.36,2.01	.716	108	7,569	0.67	0.09,4.94	.697	112	7,782
Taking folic acid supplements	0.87	0.62,1.23	.445	723	6,947	1.59	0.89,2.83	.116	727	7,161
Taking vitamins supplements	1.00	0.99,1.02	.670	1,305	6,351	1.13	0.58,1.88	.639	1,347	6,526
Taking paracetamol	1.45	1.18,1.78	4 × 10-4	4,585	3,000	0.76	0.51,1.13	.181	4,717	3,069
Taking antidepressants	0.69	0.16,2.97	.616	45	7,633	1.59	0.21,12.06	.652	47	7,844
Ever had diabetes	2.74	1.24,6.06	.013	58	7,743	4.35	1.49,12.67	.007	73	7,961
Gestational diabetes	2.57	0.90,7.36	.079	33	7,317	1.66	0.22,12.59	.626	42	7,491
Ever had hypertension	0.84	0.62,1.12	.232	1,069	6,450	0.33	0.13,0.82	.017	1,090	6,608
Gestational hypertension	1.14	0.88,1.48	.330	1,116	6,534	1.28	0.75,2.18	.369	1,127	6,748
Preeclampsia	1.07	0.54,2.10	.848	151	7,650	3.13	1.32,7.43	.010	159	7,875
Vaginal bleeding	1.05	0.81,1.37	.712	1,261	5,970	0.79	0.43,1.43	.429	1,311	6,120
Any infection	1.25	0.99,1.57	.056	1,567	5,959	0.87	0.51,1.48	.601	1,578	6,116
Ever had rheumatism	0.97	0.59,1.59	.913	318	7,198	1.29	0.51,3.24	.586	325	7,377
Ever had psoriasis	1.29	0.79,2.11	.307	262	7,223	1.15	0.41,3.18	793	268	7,404
Ever had severe depression	1.64	1.20,2.25	.002	589	6,966	0.96	0.44,2.09	0.912	572	7,171

	ADHD	*ASD*

© 2019 Leppert B et al. *JAMA Psychiatry.*

	RR	*95% CI*	*P value*	N_{yes}	N_{No}	*RR*	*95% CI*	*P value*	N_{yes}	N_{No}
BMI (pre-pregnancy)	1.01	0.99,1.04	.272	-	7,173	0.98	0.92,1.04	.561	-	7,343
Age at delivery	0.97	0.95,0.99	.004	-	7,833	1.03	0.99,1.08	.123	-	8,069
Stressful life event score	1.15	1.10,1.20	2 × 10-9	-	6,941	0.95	0.85,1.06	.325	-	7,088
Vitamin D (OHD)*	0.89	0.68,1.15	.361	-	4,546	1.11	0.66,1.88	.687	-	4,722
Selenium (Se)*	0.87	0.35,2.14	.759	-	2,418	1.73	0.29,10.41	.548	-	2,457
Mercury (Hg)	0.80	0.66,0.98	.029	-	2,330	0.89	0.64,1.14	.282	-	2,370
Cadmium (Cd)	1.40	1.06,1.83	.017	-	2,418	1.33	0.77,2.29	.314	-	2,457
Lead (Pb)	1.00	0.89,1.12	.987	-	2,416	0.85	0.64,1.14	.282	-	2,455
C-Section	1.09	0.80,1.47	.600	791	6,887	0.53	0.23,1.21	.132	820	7,083
Low birthweight (<2500g)	1.17	0.86,1.65	.416	314	7,424	1.20	0.72,2.40	.628	323	7,656
Preterm delivery (<37weeks)	1.40	0.95,2.07	.090	378	7,455	0.94	0.38,2.34	.888	400	7,669
Hypoxia	1.13	0.87,1.46	.358	1,306	3,304	0.81	0.46,1.41	.449	1,354	3,397
Low Apgar at 1 minute	1.50	1.08,2.07	.015	562	4,043	1.68	0.90,3.14	.101	583	4,166

* at 18 weeks of gestation

© 2019 Leppert B et al. *JAMA Psychiatry.*

eTable 9. Association of Maternal Polygenic Risk Scores (*P* < .05) With Maternal Alcohol Consumption, Smoking Behavior, and Stressful Life Events at Different Time Intervals During and Before Pregnancy

				ADHD			*ASD*			*Schizophrenia*		
	N (No)	*N (Yes)*	*N (Total)*	*OR*	*95% CI*	*P value*	*OR*	*95% CI*	*P value*	*OR*	*95% CI*	*P value*
Maternal alcohol consumption												
before pregnancy	491	6804	7295	0.89	0.63, 1.28	.540	0.99	0.68, 1.43	.945	1.02	0.70, 1.48	.918
during 1st trimester	3225	4051	7276	0.99	0.92, 1.06	.764	1.00	0.94, 1.08	.914	1.01	0.94, 1.08	.854
during 3rd trimester	2829	1526	4355	0.91	0.84, 0.98	.015	1.05	0.97, 1.13	.218	1.05	0.97, 1.13	.261
during pregnancy	1620	4385	6005	0.99	0.89, 1.11	.907	1.02	0.92, 1.14	.696	1.03	0.92, 1.15	.594
Maternal Smoking												
before pregnancy	5073	2257	7330	1.24	1.17, 1.32	$1x10^{-12}$	1.03	0.97, 1.10	.303	1.11	1.05, 1.18	.001
during 1st trimester	5710	1620	7330	1.25	1.17, 1.33	$1x10^{-11}$	1.04	0.98, 1.11	.184	1.14	1.04, 1.18	.001
during 3rd trimester	5630	1157	6787	1.27	1.18, 1.36	$6x10^{-11}$	1.06	0.99, 1.14	.086	1.06	0.99, 1.14	.082
during pregnancy	5205	1746	6951	1.27	1.19, 1.35	$3x10^{-13}$	1.03	0.97, 1.10	.297	1.11	1.04, 1.18	.001

© 2019 Leppert B et al. *JAMA Psychiatry.*

eTable 10. Association of Maternal Polygenic Risk Scores (PRS) (*P* < .05) With Prenatal Exposures Linked to Offspring Neurodevelopmental Disorders, Excluding Mothers Who Have Reported Taking Medication for Anxiety, Depression, Migraine, Sleeping Problem, or Other*

		PRS_{ADHD}			PRS_{ASD}			PRS_{SCZ}		
	N_{total}	*OR*	*95% CI*	*P value*	*OR*	*95% CI*	*P value*	*OR*	*95% CI*	*P value*
Smoking	4926	1.26	1.16,1.36	5×10^{-9}	1.00	0.93,1.08	.964	1.12	1.04,1.21	.003
Alcohol consumption	4208	1.00	0.88,1.13	.987	1.02	0.90,1.15	.781	1.03	0.91,1.17	.636
Binge drinking during 1st trimester	5114	1.08	0.99,1.17	.073	1.01	0.93,1.10	.826	1.04	0.96,1.13	.340
Taking iron supplements	5117	0.94	0.87,1.02	.120	0.96	0.89,1.03	.280	1.10	1.02,1.19	.018
Taking zinc supplements	5117	0.86	0.67,1.11	.248	0.93	0.72,1.21	.598	1.22	0.94,1.57	.135
Taking folic acid supplements	5110	0.93	0.84,1.03	.177	1.01	0.91,1.11	.918	1.14	1.03,1.26	.011
Taking vitamins supplements	5104	0.94	0.87,1.02	166	0.96	0.89,1.04	.360	1.11	1.02,1.20	.016
Taking paracetamol	4983	1.12	1.03,1.21	.008	1.00	0.92,1.08	.909	0.98	0.91,1.06	.654
Ever had diabetes	5399	1.37	1.01,1.85	.041	1.02	0.75,1.37	.909	0.88	0.65,1.19	.395
Gestational diabetes	4813	1.59	1.03,2.46	.037	1.07	0.70,1.63	.754	0.98	0.63,1.51	.921
Ever had hypertension	4919	1.05	0.96,1.14	.331	1.02	0.94,1.12	.613	1.08	0.98,1.18	.106
Gestational hypertension	5281	0.97	0.90,1.06	.541	1.00	0.92,1.09	.955	1.03	0.95,1.12	.499
Preeclampsia	5399	1.14	0.95,1.38	.159	1.23	1.02,1.48	.028	1.08	0.90,1.30	.415
Vaginal bleeding	4836	1.05	0.96,1.14	.267	1.02	0.94,1.10	.720	1.08	1.00,1.18	.058
Any infection	4839	1.10	1.02,1.19	.018	1.05	0.97,1.13	.224	1.02	0.94,1.10	.652
Ever had rheumatism	4941	0.95	0.82,1.11	.523	0.96	0.82,1.11	.577	1.00	0.85,1.16	.966
Ever had psoriasis	4929	1.02	0.88,1.19	.791	1.10	0.95,1.28	.210	1.03	0.88,1.20	.693
Ever had severe depression	4965	1.19	1.06,1.34	.003	1.04	0.93,1.16	.546	1.26	1.13,1.42	6×10^{-5}

* other medication that was not used for nausea, heartburn, vomiting, infection, pain, allergies, skin condition, bleeding, piles, constipation and cough
ADHD attention deficit/hyperactivity disorder, *OR* odds ratio, *CI* confidence interval

© 2019 Leppert B et al. *JAMA Psychiatry.*

eTable 10 (continued)

		PRS$_{ADHD}$				PRS$_{ASD}$				PRS$_{SCZ}$		
	N_{total}	*β*	*95% CI*	*P value*		*β*	*95% CI*	*P value*		*β*	*95% CI*	*P value*
BMI (pre-pregnancy)	4719	0.25	0.14,0.35	5×10^{-6}		0.01	-0.09,0.11	.850		-0.20	-0.31,-0.09	2×10^{-4}
Age at delivery	5454	-0.44	-0.57,-0.31	1×10^{-11}		0.09	-0.03,0.22	.143		0.08	-0.05,0.20	.249
Stressful life event score in 1st trimester	4847	0.04	-0.03,0.11	.223		0.07	0.01,0.14	.033		0.04	-0.03,0.11	.257
Vitamin D (OHD)*	3486	0.00	-0.01,0.02	.882		-0.01	-0.02,0.01	.340		0.01	-0.01,0.02	.292
Selenium (Se)*	1903	-0.01	-0.02,0.00	.005		-0.004	-0.01,0.004	.315		0.00	-0.01,0.01	.745
Mercury (Hg)	1838	-0.06	-0.11,-0.01	.023		0.02	-0.03,0.07	.511		0.04	-0.02,0.09	.169
Cadmium (Cd)	1903	0.06	0.03,0.08	2×10^{-5}		0.01	-0.02,0.04	.440		0.03	0.00,0.05	.047
Lead (Pb)	1903	-0.01	-0.07,0.06	.799		0.03	-0.04,0.09	.430		-0.01	-0.08,0.05	.702

ADHD attention deficit/hyperactivity disorder, β beta coefficients, *CI* confidence interval, * log transformed

© 2019 Leppert B et al. *JAMA Psychiatry.*

eTable 10 (continued)

		PRS_{ADHD}				PRS_{ASD}				PRS_{SCZ}		
	N_{total}	*OR*	*95% CI*	*P value*		*OR*	*95% CI*	*P value*		*OR*	*95% CI*	*P value*
C-Section	5326	1.03	0.94,1.13	.519		1.04	0.95,1.14	.436		0.96	0.87,1.05	.359
Low birthweight (<2500g)	5378	0.84	0.41,1.74	.640		0.99	0.52,1.86	.967		0.97	0.52,1.81	.912
Preterm delivery (<37weeks)	5454	1.03	0.91,1.16	.671		0.98	0.87,1.11	.800		0.98	0.87,1.11	.754
Hypoxia	3178	1.12	1.02,1.23	.018		1.07	0.98,1.17	.144		1.06	0.96,1.16	.248
Low Apgar at 1 minute	3177	1.16	1.04,1.30	.011		1.08	0.96,1.21	.191		1.04	0.93,1.16	.509
Low Apgar at 5 minutes	3173	1.28	0.90,1.81	.167		1.24	0.88,1.75	.222		0.84	0.59,1.20	.344
Breast fed their child	5009	0.86	0.73,1.01	.071		1.03	0.88,1.21	.703		1.05	0.89,1.24	.545

ADHD attention deficit/hyperactivity disorder, *OR* odds ratio, *CI* confidence interval

© 2019 Leppert B et al. *JAMA Psychiatry.*

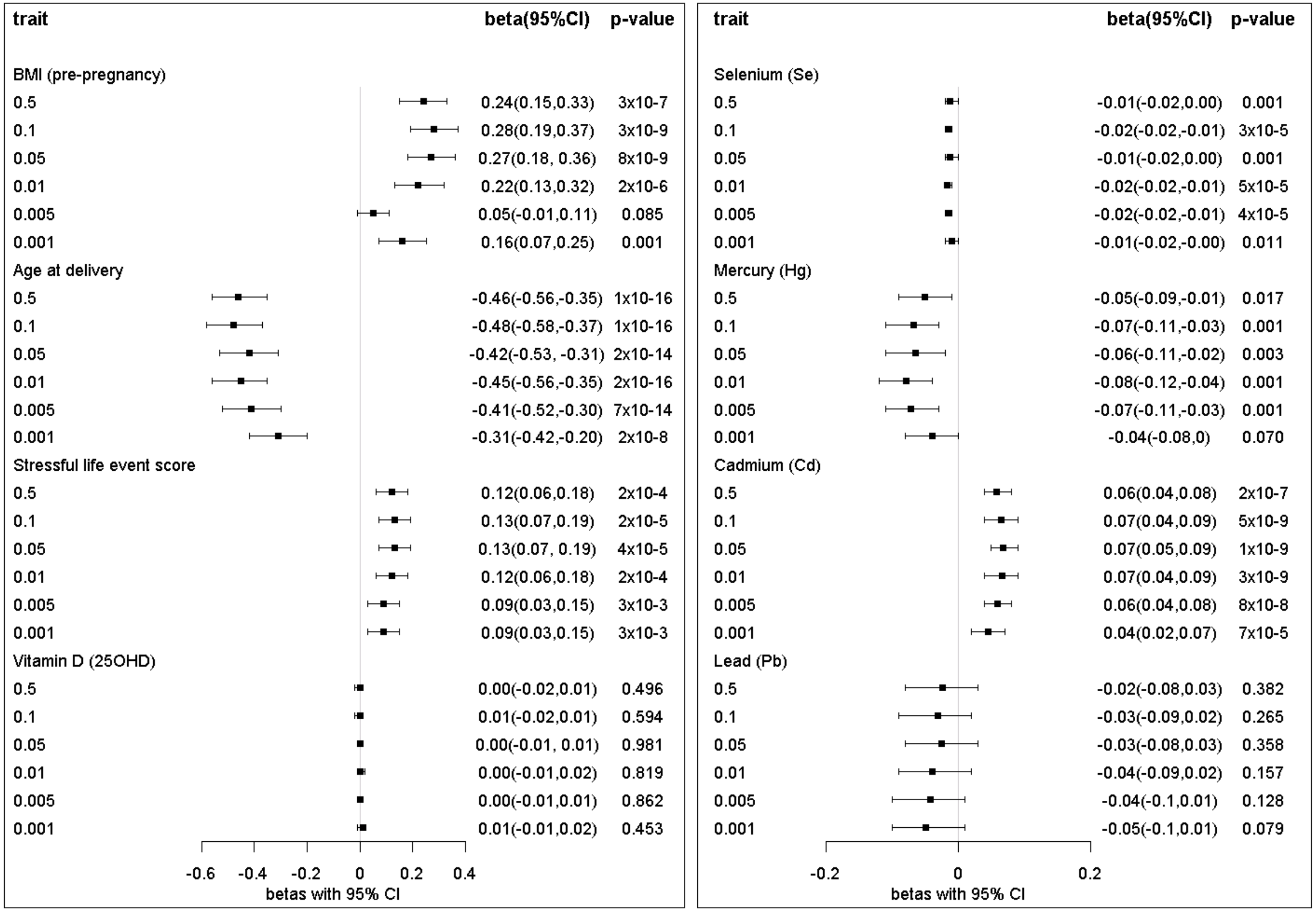

eFigure 1. Association of Maternal Polygenic Risk Scores for ADHD at Different *P* Value Thresholds With Prenatal and Perinatal Exposures Linked to Offspring Neurodevelopmental Disorders

© 2019 Leppert B et al. *JAMA Psychiatry.*

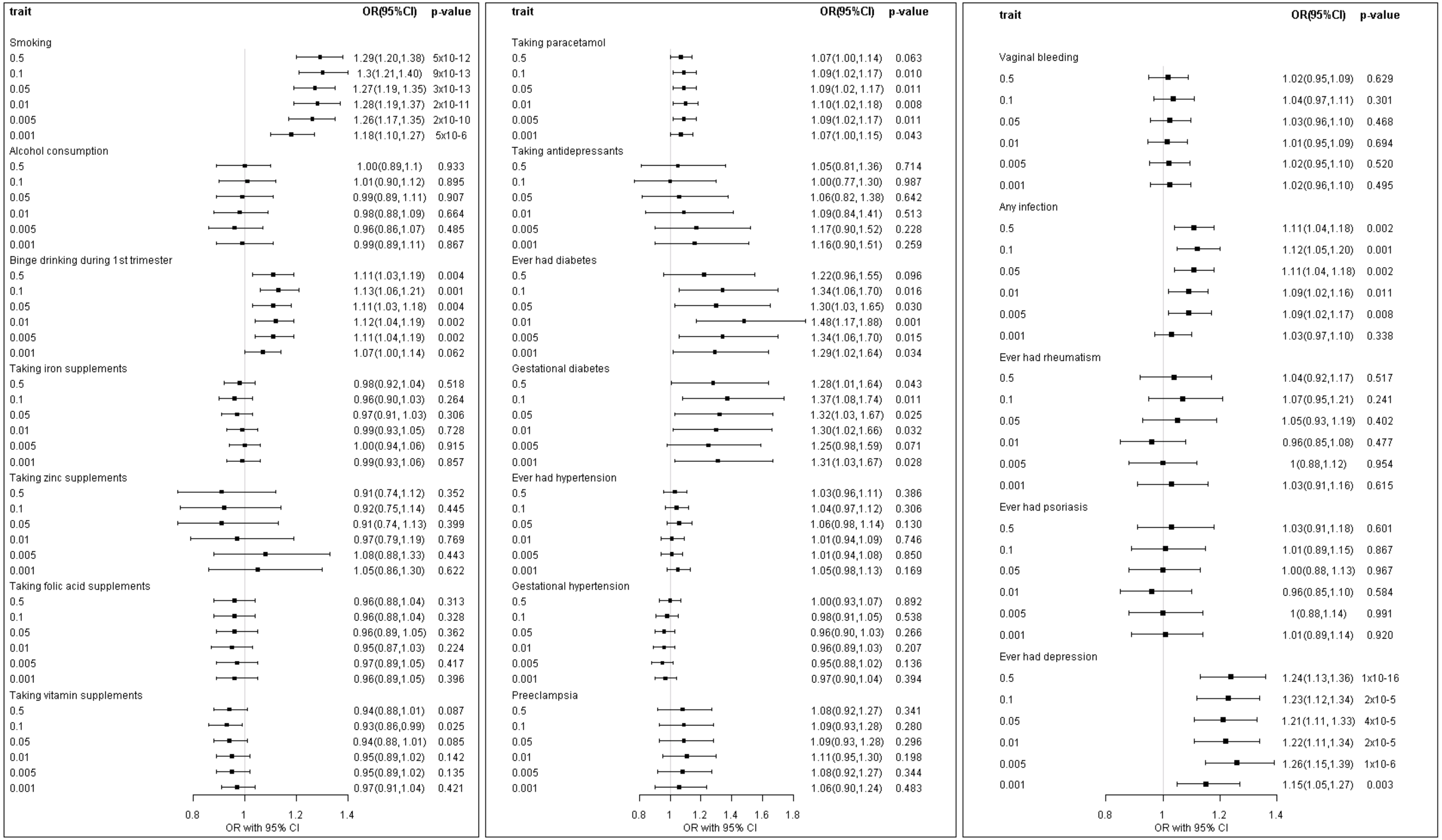

eFigure 2. Association of Maternal Polygenic Risk Scores for ADHD at Different *P* Value Thresholds With Prenatal and Perinatal Exposures Linked to Offspring Neurodevelopmental Disorders

© 2019 Leppert B et al. *JAMA Psychiatry.*

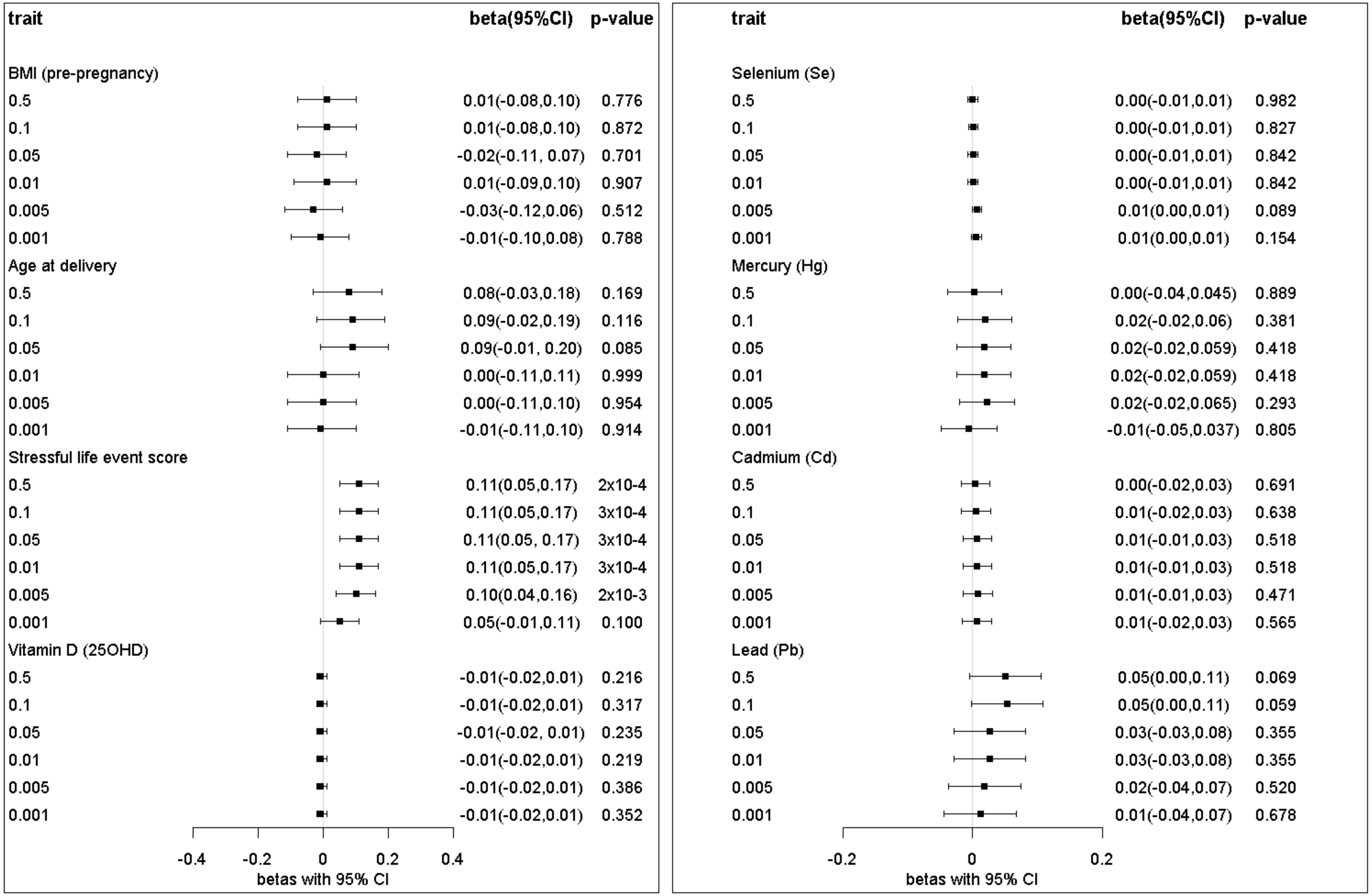

eFigure 3. Association of Maternal Polygenic Risk Scores for Autism at Different *P* Value Thresholds With Prenatal and Perinatal Exposures Linked to Offspring Neurodevelopmental Disorders

© 2019 Leppert B et al. *JAMA Psychiatry.*

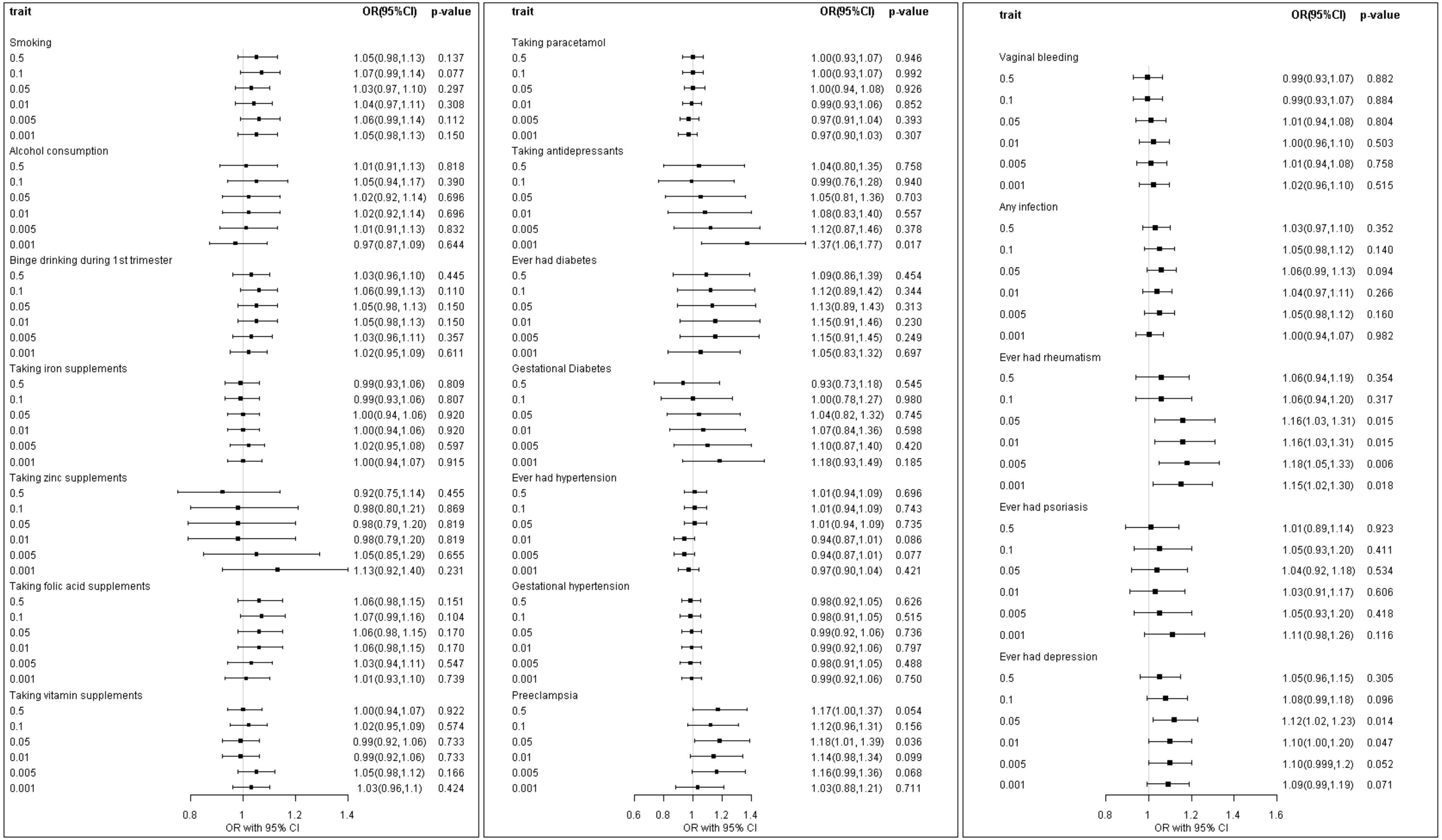

eFigure 4. Association of Maternal Polygenic Risk Scores for Autism at Different *P* Value Thresholds With Prenatal and Perinatal Exposures Linked to Offspring Neurodevelopmental Disorders

© 2019 Leppert B et al. *JAMA Psychiatry.*

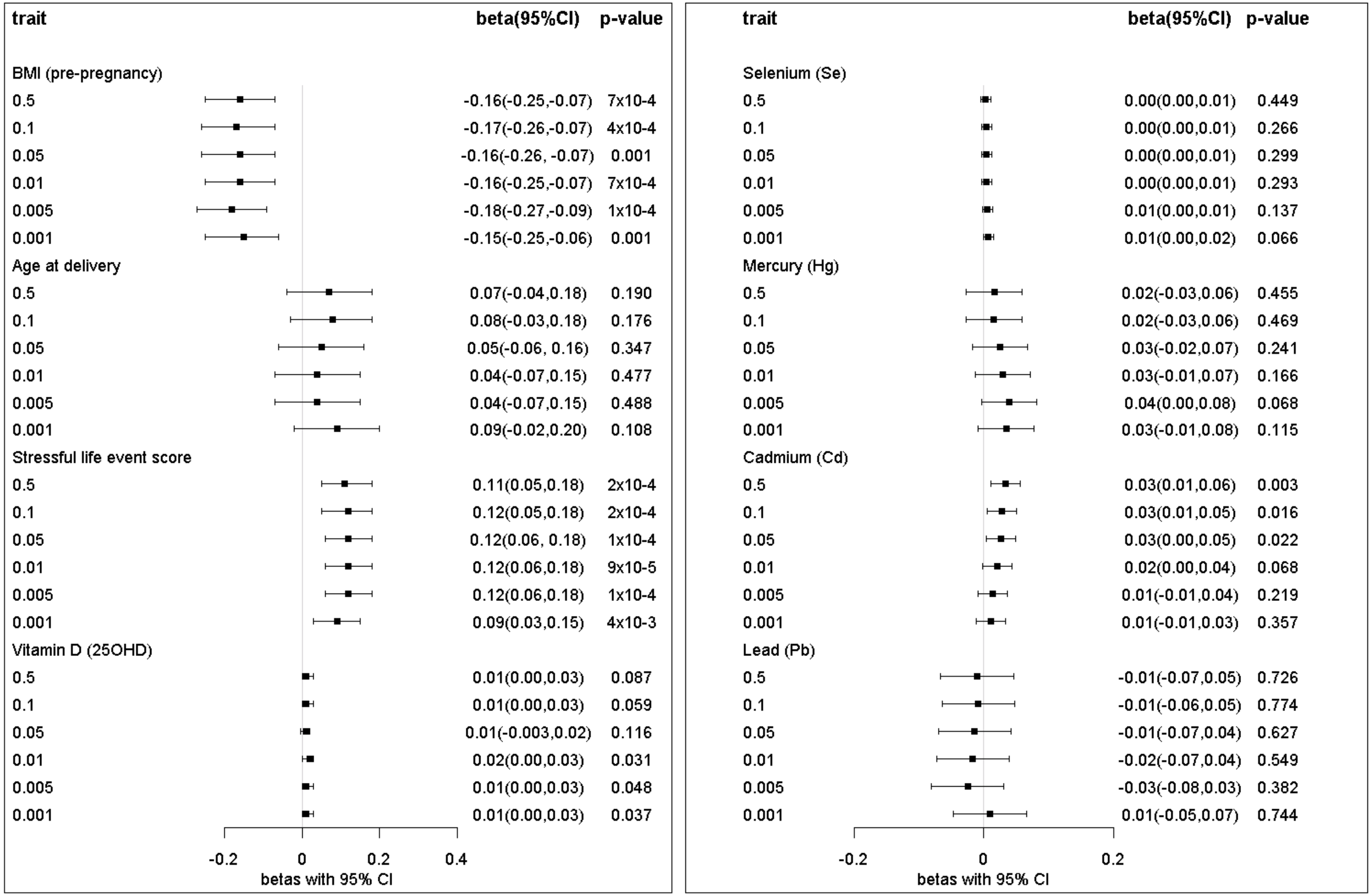

eFigure 5. Association of Maternal Polygenic Risk Scores for Schizophrenia at Different *P* Value Thresholds With Prenatal and Perinatal Exposures Linked to Offspring Neurodevelopmental Disorders

© 2019 Leppert B et al. *JAMA Psychiatry.*

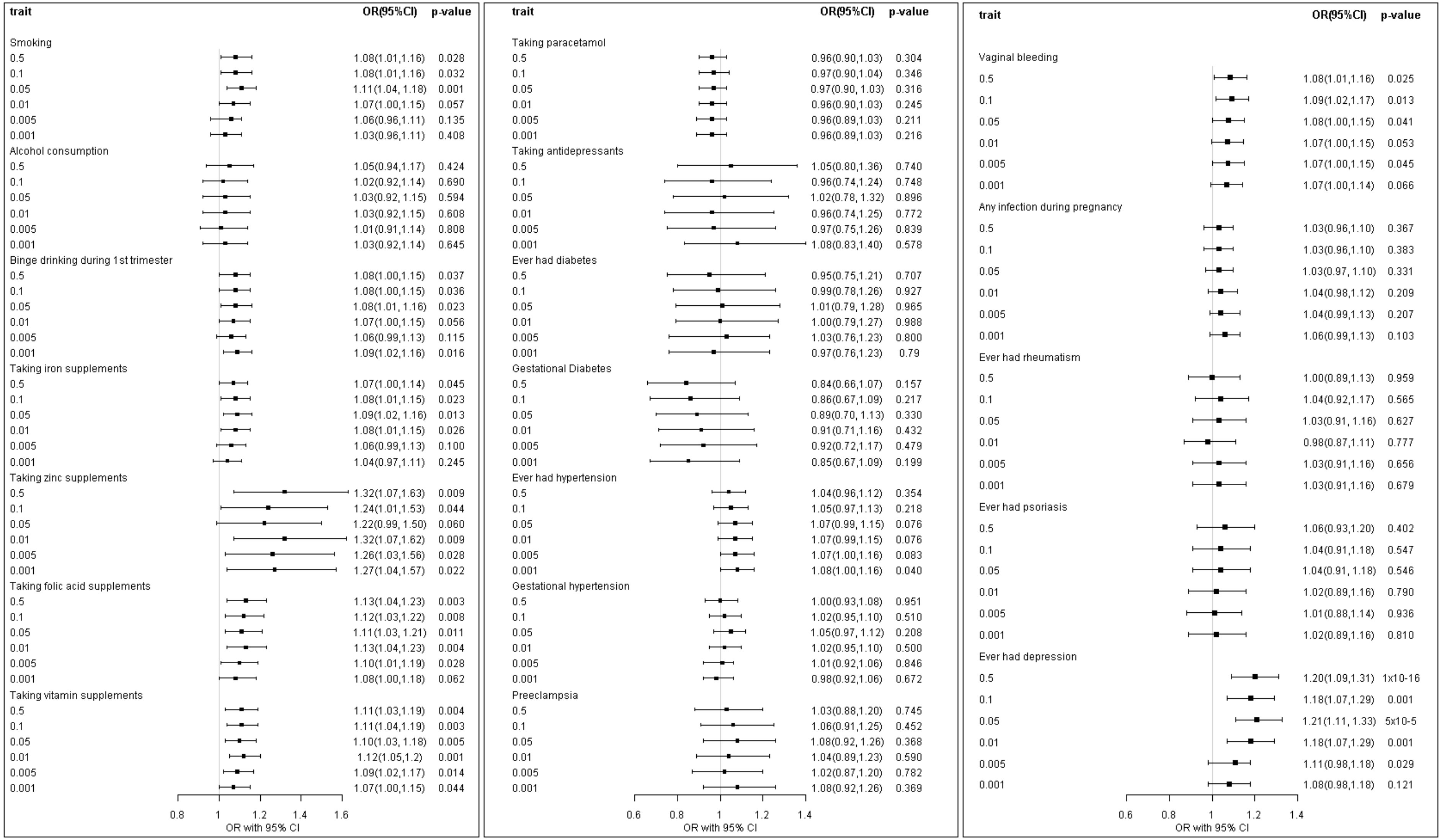

eFigure 6. Association of Maternal Polygenic Risk Scores for Schizophrenia at Different *P* Value Thresholds With Prenatal and Perinatal Exposures Linked to Offspring Neurodevelopmental Disorders

© 2019 Leppert B et al. *JAMA Psychiatry.*
